# A 23-year-old man with left lung atelectasis treated with a targeted segmental recruitment maneuver: a case report

**DOI:** 10.1186/s13256-020-02409-6

**Published:** 2020-06-24

**Authors:** Alen Protić, Matej Bura, Kazimir Juričić

**Affiliations:** Department of Anesthesiology and ICU, University Hospital Rijeka, Tome Strizica 3, 51 000 Rijeka, Croatia

**Keywords:** Lung atelectasis, Segmental recruitment, Prolonged recruitment

## Abstract

**Background:**

Lung atelectasis are nonventilated parts of lung tissue and occur as a result of the collapse of the pulmonary parenchyma (alveoli). Various therapeutic procedures for inflating the collapsed pulmonary parenchyma, such as bronchial aspiration and/or standard recruitment maneuvers, are not always successful.

**Case presentation:**

We report a case of a 23-year-old Croatian man with a parapharyngeal abscess on the left side of the neck with spreading of infection in the mediastinum and left side of the thorax and consequent major atelectasis of the left lung. The patient was mechanically ventilated. We decided to apply a new method in which a pulmonary artery catheter was placed (guided by bronchoscope) on the entrance to the lower left bronchus. The pulmonary artery catheter balloon was inflated to achieve bronchial closure. Using another respirator, we ventilated the affected lobe separately with continuously high pressure of 30 cmH_2_O. After 30 minutes, we removed the pulmonary artery catheter from the lower left bronchus and placed it in the upper left bronchus and repeated the procedure. Our method allowed a significantly longer duration (30 minutes) of continuously high pressure of 30 cmH_2_O separately to only one of the total of five lobes of the lungs while the other four lobes were simultaneously ventilated continuously with protective ventilation mode.

**Conclusion:**

Use of a pulmonary artery catheter and two respirators in our patient’s case proved to be a successful method for recruiting the atelectatic lung while maintaining protective ventilation of the lung segments without atelectasis.

## Background

Lung atelectasis are nonventilated parts of lung tissue and occur as a result of the collapse of the pulmonary parenchyma (alveoli). Usually, it appears in the lower and posterior portions of the lung due to a long duration in supine position, which can enhance an inflammatory process in the lung and result in pneumonia [1]. Lung atelectasis in clinical practice can be treated with bronchoaspiration and continuous positive airway pressure (CPAP) during spontaneous breathing or positive end-expiratory pressure (PEEP) during mechanical ventilation [2]. Both methods have an effect on the entire pulmonary parenchyma, including nonatelectatic parts of the lung. By increasing the basic PEEP level to 10 or 12 cmH_2_O in mechanically ventilated patients, trying to prevent atelectasis, the side effects of overstretching unaffected alveoli and increasing intrathoracic pressure also occur. Barotrauma and volutrauma of the nonatelectatic lung parenchyma may result, as well as hemodynamic instability due to decreased cardiac output [3, 4]. A more invasive approach to resolving lung tissue atelectasis in the last 15 years has been the recruitment maneuver (inspiratory hold) for 30 to 40 seconds with a positive pressure of 30 to 40 cmH_2_O, where open alveoli are inflated more extensively and the effect on closed alveoli is questionable.

## Case presentation

A 23-year-old Croatian man without any significant past medical history was admitted to our intensive care unit (ICU) due to a parapharyngeal abscess on the left side of the neck with spreading of infection in the mediastinum and the left side of the thorax. Urgent surgery was performed with incision of the parapharyngeal abscess, neck dissection, and left side thoracotomy with incision and drainage of the mediastinum and thorax. In the postoperative period in the ICU, the patient was sedated and mechanically ventilated with antibiotic therapy according to microbiological findings (blood, urine, bronchoalveolar lavage, and tissue sample taken during surgery). After the first surgery, *Streptococcus mitis* was isolated from the parapharyngeal abscessus. In the second surgery, *Staphylococcus* sp. was isolated from the mediastinal and neck swab wounds. From the beginning (upon admission to the ICU), the patient was treated with meropenem and linezolid. During the second week of treatment, he started to develop a nosocomial infection of the lung caused by multiresistant *Pseudomonas aeruginosa*, which was treated with ceftolozane/tazobactam 3 × 3 g intravenously. In the next 10 days, control computed tomographic (CT) scans of the neck and chest showed progression of mediastinal infiltrates and the formation of organized pleural effusion on the left side that required additional surgery. Due to atelectasis of the left lung that persisted during the second week of treatment in the ICU, bronchoscopy with bronchoaspiration and recruitment maneuvers were performed several times. Residual pleural effusions were drained several times before and after weaning procedures with a small-bore pleural catheter and Seldinger technique. On the 30th day of the patient’s illness, sedation was stopped, and the patient awoke promptly. He was hemodynamically stable and ready for weaning, which was successfully done in the next 24 hours. When the patient was extubated and started to breathe spontaneously, we enhanced his active physical therapy in combination with a cough assist device (CoughAssist E70; Philips Respironics, Hamburg, Germany). After the weaning procedure, the patient was fully conscious and hemodynamically stabile with blood pressure 115/70 mmHg, heart rate of 86 beats/minute, and body temperature of 36.7 °C. The patient was spontaneously breathing with oxygen saturation (SpO_2_) of 94%, fraction of inspired oxygen (FiO_2_) of 50%, partial pressure of oxygen (PaO_2_) of 9.19 kPa, carbon dioxide pressure (pCO_2_) of 5.7 kPa, and PaO_2_/FiO_2_ ratio of 138, but almost no rising of the left side of the thorax and no breath sounds on the same side.

**Fig. 1:**
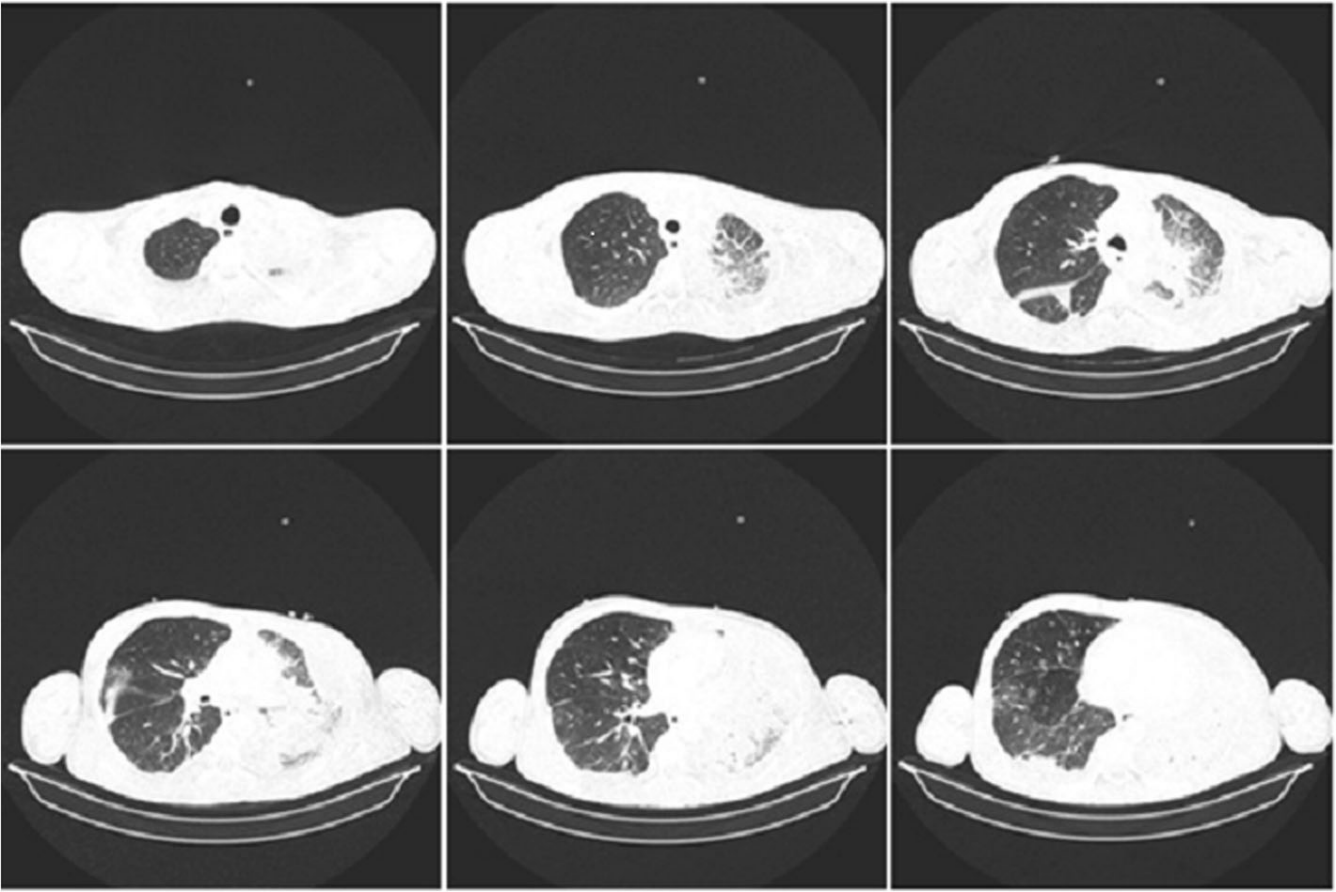
Computed tomographic scan of the chest of the patient on the 34th day of his stay in the intensive care unit

We performed lung ultrasound, which showed atelectasis of the major part of the left lower lobe and the posterior part of the upper lobe on the 34th day of the patient’s stay in the ICU. We used electrical impedance tomography (Dräger PulmoVista 500; Dräger, Lübeck, Germany) as additional diagnostic support for the ultrasound, which confirmed reduced air entrance in the major part of the left lung. Finally, according to local protocol, we performed a CT scan of the chest, which confirmed previous findings of complete atelectasis of the left lower lobe and major atelectasis of the left upper lobe (Fig. 1).

We decided to apply a new method whereby we used different experiences of single-lung ventilation respecting all basic principles of various types of mechanical ventilation. Our trial, which we named “targeted segmental recruitment,” was one of the last attempts to avoid the proposed life-threatening surgical reduction of the lung parenchyma in our young patient. His Acute Physiology and Chronic Health Evaluation II (APACHE II) score upon admission was 7, and his Sequential Organ Failure Assessment (SOFA) score upon admission was 6. On the day before we performed the segmental recruitment maneuver, his APACHE II score was 12, and his SOFA score was 6. His procalcitonin level on the day before segmental recruitment maneuver was 0.171 μg/L.

**Fig. 2:**
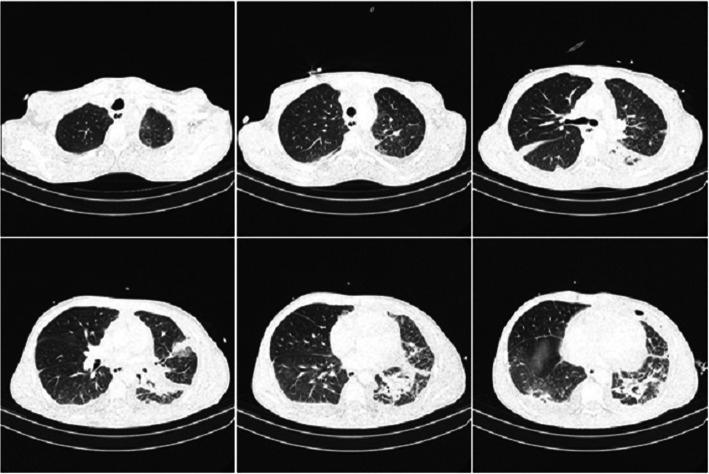
Computed tomographic scan of the chest of the patient after targeted segmental recruitment (36th day of stay in the intensive care unit)

The patient was analgosedated, intubated with the Univent 8.5-mm tube (tube with integrated endobronchial blocker; Vitaid, Lewiston, NY, USA), and mechanically ventilated. We removed the endobronchial blocker and replaced it with a pulmonary artery (PA) catheter. With the help of a bronchoscope, using a loop through the working channel of the bronchoscope, the PA catheter was placed in the entrance to the lower left bronchus, and the PA catheter balloon was inflated to achieve bronchial closure. Using the appropriate connectors, we connected the PA catheter to the second ventilator (Dräger Evita 2) and applied 30 cmH_2_O of pressure of the 30% oxygenated air in CPAP ventilation mode for 30 minutes. The decision for the pressure of 30 cmH_2_O was made on the basis of the fact that pressures higher than 35 cmH_2_O are associated with barotrauma and the clinical appearance of pneumothorax [5]. The right lung and the upper lobe of the left lung were ventilated the entire time by controlled mechanical ventilation with protective ventilation parameters using the Dräger Evita XL ventilator. After 30 minutes, we removed the PA catheter from the lower left bronchus and placed it in the upper left bronchus with the described procedure and repeated the CPAP maneuver. Again, the right lung and the lower lobe of the left lung were simultaneously ventilated by controlled mechanical ventilation with protective ventilation parameters. Within the next 12 hours of the performed procedure, the patient was awakened and extubated. After the targeted segmental recruitment, he was breathing spontaneously with improved clinical parameters as well as better rising of the left side of the thorax with audible respiratory sounds on the left side. The PulmoVista 500 monitoring was applied continuously, and better ventilation of the left lung was noted in the first hours after the procedure. In the next 48 hours, we applied the CoughAssist device to the patient, and he was encouraged to cough. After 48 hours (36th day of treatment), a CT scan of the thorax was performed, indicating significantly better ventilation of the left lung (Fig. 2). During and after the procedure, SpO_2_, end-tidal carbon dioxide (EtCO_2_), invasive blood pressure, and pulse were monitored. The patient did not have any significant decrease in SpO_2_ or increase in EtCO_2_, nor did he have hemodynamic instability or changes in heart rhythm. Before and after the procedure, arterial blood gas analyses were performed as part of routine laboratory processing (Table 1). The patient was discharged to home 49 days after admission, and he was mobile on his own with stabile hemodynamic and respiratory status and without signs of infection.

**Table 1:** Findings of blood gas analysis before extubation (30th day), before (34th day) and after (35th and 36th days) targeted segmental recruitment

	30th day	34th day		35th day	36th day
FiO_2_ (%)	40	50		40	40
pH	7.44	7.39		7.44	7.44
PaO_2_ (kPa)	9.9	9.19		12.3	14.5
PaCO_2_ (kPa)	4.6	5.7	Targeted segmental recruitment	5.2	5.1
PaO_2_/FiO_2_	187.25	137.86		230.64	271.90
SpO_2_ (%)	95.2	94.2		97.2	98.2
Lactate (mmol/L)	0.9	0.8		0.8	0.7
CRP	277.2	101.6		96.1	97.3

*Abbreviations: CRP* C-reactive protein, *FiO*_*2*_ Fraction of inspired oxygen, *PaO*_*2*_ Partial pressure of arterial oxygen, *PaCO*_*2*_ Carbon dioxide pressure, *SpO*_*2*_ Oxygen saturation

## Discussion

The collapse of the pulmonary parenchyma is a problem that became more visible when diagnostic methods such as CT scanning and ultrasound became more commonly used in clinical practice. In the past few decades, many good ideas have found useful application, such as increasing the basic PEEP level, prone positioning of the patient, recruitment maneuver with inspiratory hold, and others. The recruitment maneuver has become standard practice in many ICUs all over the world, despite a well-known effect being overstretching of unaffected alveoli with a questionable effect on collapsed alveoli. A study that used a recruiting maneuver in patients with pneumonia combined with acute respiratory distress syndrome or the need for vasopressors showed increased mortality, which confirmed the potential harm of recruiting maneuvers [6]. Unilateral recruitment using an endobronchial blocker has been described in the literature as a successful method of treating atelectasis [7]. The endobronchial blocker was used to protect the left lung while recruitment of the right lung atelectasis was applied for 3 minutes. In this case report, step-forward was done regarding duration of the recruitment maneuver, despite the fact that healthy lung was not ventilated in that period.

In our clinical practice, we often use the CPAP recruitment maneuver, usually with 40 cmH_2_O pressure for 40 seconds if the patient does not have any hemodynamic instability. If hemodynamic instability is present, we shorten the period of inflation to 20 seconds. Our patient was a healthy person with no comorbidities and showed no emphysematous or other changes besides pneumonic infiltration in the lung parenchyma, which can cause barotrauma to the lungs. In the literature, recruitment maneuvers with pressure greater than 30 cmH_2_O are often described. In cases of chronic changes in the lung, we will surely reduce the time of the inflation. After the extubation, we usually encourage secretion removal with aggressive physical therapy and the CoughAssist device. Bronchoaspiration before extubation is our routine procedure in these patients. Therefore, we concluded that we had a patient who had undergone many repeated therapeutic procedures, all with the purpose of inflating the left lung (bronchial aspiration, standard recruitment maneuvers, thoracic drainage), and surgical lung resection became an option for treatment. Therapeutic options according to the new idea of targeted segmental recruitment as a lifesaving procedure were discussed with the patient’s mother, and she signed the informed consent.

Our method allowed a significantly longer duration (30 minutes) of continuously high pressure of 30 cmH_2_O separately to only one of the five total lobes of the lungs, which meant significantly less increase in the total intrathoracic pressure in comparison with a classic recruitment maneuver. The targeted segmental recruitment procedure significantly reduces the risk of barotrauma of healthy alveoli as well as the negative effect on the hemodynamic stability of patients compared with conventional recruitment maneuvers.

## Conclusion

Targeted segmental recruitment with the help of a PA catheter and the use of two respirators in our patient’s case proved to be a successful method for recruiting the atelectatic lung while maintaining protective ventilation of the lung segments without atelectasis.

## Data Availability

The datasets used and/or analyzed during the current study are available from the corresponding author on reasonable request. All data generated or analyzed during this study are included in this published article.

## Abbreviations

APACHE II: Acute Physiology and Chronic Health Evaluation II
CPAP: Continuous positive airway pressure
CT: Computed tomographic
EtCO_2_: End-tidal carbon dioxide
ICU: Intensive care unit
PA: Pulmonary artery
PEEP: Positive end-expiratory pressure
SOFA: Sequential Organ Failure Assessment
